# Omega‐3 Fatty Acids Supplementation and Neuroprotection, Inflammation, Fatigue, and Physical Activity in Multiple Sclerosis: A Randomized Controlled Trial

**DOI:** 10.1002/fsn3.70884

**Published:** 2025-09-01

**Authors:** Sahar Golabi, Tahereh Robahat, Nastaran Madjdinasab, Naser Kamyari, Mahshid Naghashpour

**Affiliations:** ^1^ Department of Medical Physiology, School of Medicine Abadan University of Medical Sciences Abadan Iran; ^2^ Student Research Committee Abadan University of Medical Sciences Abadan Iran; ^3^ Musculoskeletal Rehabilitation Research Center Ahvaz Jundishapur University of Medical Sciences Ahvaz Iran; ^4^ Department of Biostatistics and Epidemiology, School of Health Abadan University of Medical Sciences Abadan Iran; ^5^ Department of Nutrition, School of Medicine Abadan University of Medical Sciences Abadan Iran

**Keywords:** brain‐derived neurotrophic factor, clinical trial, high‐sensitivity C‐reactive protein, multiple sclerosis, omega‐3 fatty acids, placebo

## Abstract

Omega‐3 fatty acids have neuroprotective properties. The present study aimed to evaluate the influence of omega‐3 fatty acids supplementation on the serum levels of brain‐derived neurotrophic factor (BDNF), high‐sensitivity C‐reactive protein (hs‐CRP), physical activity, and chronic fatigue in patients with multiple sclerosis (MS). In a double‐blind, placebo‐controlled trial, 68 MS patients were randomly assigned to the intervention group receiving omega‐3 fatty acids soft gels (1000 mg) twice a day for 12 weeks and the placebo group similarly taking paraffin soft gels. Serum concentrations of BDNF and hs‐CRP were assessed before and after the intervention. Chronic fatigue, physical activity, and dietary intake of omega‐3 fatty acids were evaluated. Initial comparisons between the intervention and control groups showed no significant differences for BDNF and hs‐CRP levels (*p* = 0.321 and *p* = 0.996, respectively). Following the intervention, both groups showed a significant increase in their BDNF levels (*p* < 0.001). However, there were no significant differences in post‐intervention levels of BDNF and hs‐CRP serum levels between groups (*p* = 0.427, *p* = 0.695, respectively). This finding was further supported by the Quade nonparametric analysis of covariance, adjusted for baseline values, which indicated that omega‐3 fatty acid supplementation had no significant effect on BDNF or hs‐CRP levels in patients with MS (*p* = 0.644 and *p* = 0.533, respectively). After the intervention, hs‐CRP changes were 1.5 folds greater in female patients than in male patients (*p* = 0.005). Additionally, no significant differences were observed between the intervention and control groups in baseline or post‐intervention physical activity levels or chronic fatigue (*p* > 0.05), suggesting that omega‐3 fatty acid supplementation did not influence physical activity or fatigue. Omega‐3 fatty acids supplementation has no significant effect on BDNF and hs‐CRP serum levels, fatigue, or physical activity capacity among MS patients. However, gender‐specific differential hs‐CRP response suggests a possible sex‐related anti‐inflammatory effect, which deserves further investigation.

## Graphical abstract

In this randomized, placebo‐controlled trial, ommega‐3 and placebo increased BDNF levels with no significantly change found in BDNF or hs‐CRP serum levels when adjusting for baseline levels. In conclusion, omega‐3 supplementation has no determinate impacts on BDNF levels, fatigue, or physical activity capacity in MS patients. However, gender‐specific differential hs‐CRP response suggests a possible sex‐related anti‐inflammatory effect which deserves further investigation.

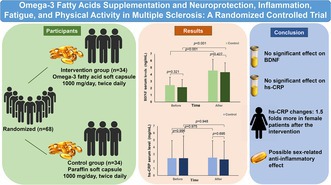

## 1 Introduction

Multiple sclerosis (MS) is an autoimmune and inflammatory disease in which the myelin of the central nervous system (CNS) is attacked by the body's immune system (Nejati et al. 2016; Sospedra and Martin 2016). Women are nearly twice as likely as men to develop multiple sclerosis (MS) (McAlpine and Compston 2005). The disease is marked by myelin degradation and resulting scar formation, which contribute to physical impairments and cognitive dysfunction. Motor disability in individuals with MS is commonly assessed using the Expanded Disability Status Scale (EDSS), a standardized tool that evaluates both physical and psychological aspects of the condition (Kurtzke 1983).

The most common symptoms of MS include paresthesia, diplopia, vision loss, fatigue, numbness or limb weakness, bowel or bladder dysfunction, ataxia, spasms, and cognitive modifications (Gaby 2013). Experienced by more than 80% of MS patients, fatigue is regarded as the most disabling symptom, significantly affecting quality of life (Freal et al. 1984). Previous studies suggest that nonpharmacological interventions for MS‐related fatigue are preferred due to lower costs, fewer side effects, and greater effectiveness compared to drug treatments (Asano and Finlayson 2014; Rahimlou, Hosseini, et al. 2022; Rahimlou, Nematollahi, et al. 2022).

MS encompasses various forms. In relapsing–remitting MS (RRMS), 85% of cases experience disease episodes followed by recovery phases. A steady and gradual worsening of symptoms marks secondary progressive MS (SPMS). Fewer than 10% of patients experience primary progressive MS (PPMS) (Benedict and Fazekas 2009; Calabrese et al. 2013; Calabrese et al. 2009; Correale et al. 2012; Hawkins and McDonnell 1999; Portaccio et al. 2009; Stern et al. 2014).

First‐line treatment to prevent relapses in RRMS patients includes various types of beta‐interferons. However, the biological mechanisms behind their therapeutic effects are not fully understood. Among these, Betaferon (IFNβ‐1b) has shown the greatest impact in increasing brain‐derived neurotrophic factor (BDNF) compared to Rebif and Avonex (Majdinasab et al. 2019). Moreover, elevated serum levels of high‐sensitivity C‐reactive protein (hs‐CRP) during the active phase of the disease can serve as supportive evidence for the diagnosis of MS (Ji et al. 2016).

Environmental factors may play a role in triggering MS (Ascherio 2013). Dietary strategies, including nutrition and targeted nutritional interventions, play a significant role in the pathogenesis of MS. By modulating oxidative stress and immune function, these approaches have shown promising effects on disease activity and progression (Stoiloudis et al. 2022).

A dietary approach emphasizing nutritional supplementation has been proposed to help reduce the side effects of immune‐modulating therapies and relieve symptoms of chronic fatigue syndrome associated with MS (Labuschagnea and Blaauw 2018). One such strategy involves the use of omega‐3 (n‐3) fatty acids—a vital group of unsaturated fats essential for numerous physiological functions. Omega‐3 fatty acids have been associated with a wide range of health benefits, including protective effects against *Helicobacter pylori* infection (Khandouzi et al. 2015), spinal cord injury (Sabour et al. 2015; Turczyn et al. 2022), diabetes mellitus (Delpino et al. 2022), hyperlipidemia (Mansoori et al. 2015; Shidfar et al. 2008). Omega‐3 (n‐3) fatty acids may also benefit by diminishing CRP concentration in patients with cardiometabolic disorders (Amlashi et al. 2025).

The most frequent omega‐3 fatty acids in our diet include alpha‐linolenic acid (ALA), eicosapentaenoic acid (EPA), and docosahexaenoic acid (DHA) (Kim et al. 2014). ALA is commonly found in foods such as walnuts, chia seeds, specific beans, various vegetables, soybean oil, canola oil, flaxseed, and olive oil (Kris‐Etherton et al. 2000). EPA and DHA are primarily sourced from fish like salmon, as well as fish oils and supplements derived from fish oil. A wealth of research has highlighted the protective effects of omega‐3 fatty acids against neurodegenerative diseases by modulating nerve activity, influencing immune cell behavior, and exhibiting strong anti‐inflammatory effects (Heng et al. 2015; Lobo et al. 2016; Lu et al. 2013). Omega‐3 fatty acids help protect neurons from oxidative damage by producing neuroprotective compounds derived from DHA. Additionally, they influence the expression of neuroprotective genes, including the anti‐apoptotic *Bcl‐2* gene (Bazan 2006).

Omega‐3 fatty acids have an important function in MS. They encourage remyelination and lower inflammation, leading to better outcomes for those with MS (Siegert et al. 2017). Systematic reviews on the influence of omega‐3 fatty acids, including EPA, DPA, and DHA, regarding the progression of MS indicate that fish oil and omega‐3 fatty acids supplements may improve the quality of life for patients. These beneficial effects are associated with their influence on inflammatory markers, the activity of glutathione reductase, reduced relapse rates, and the achievement of optimal ratios between omega‐6 and omega‐3 fatty acids (AlAmmar et al. 2021). Moreover, EPA and DHA, which are present in high concentrations in the brain, are reduced in MS patients (Prospective Studies et al. 2007). Omega‐3 fatty acids (or omega‐3 polyunsaturated fatty acids [n‐3 PUFAs]) greatly influence gene expression. By inhibiting NF‐kB (a transcription factor that stimulates inflammatory genes), omega‐3 fatty acids suppress lipogenesis‐related SREBP‐1c (sterol regulatory element‐binding protein 1c) and LXR (liver‐x receptor), and decrease MMP‐9 levels in patients with MS and inflammatory processes, resulting in reducing the risk of or improving the condition of MS (AlAmmar et al. 2021).

Studies also suggest that reducing fats, calories, and carbohydrates while increasing omega‐3 fatty acids reduces inflammatory processes, decreases MS risk, and enhances recovery in MS patients (Bjornevik et al. 2017, 2019; Esposito et al. 2018; Riccio 2011). Animal studies indicate that omega‐3 fatty acid intake enhances remyelination in MS patients (Siegert et al. 2017). Additionally, studies suggest that areas with diets rich in omega‐3 fatty acids report lower incidences of MS (Dopkins et al. 2018).

Dietary interventions can elicit placebo effects shaped by an individual's prior experiences, expectations, and reactions to specific foods, as well as cultural beliefs, personal attitudes toward diet, sensory enjoyment, taste preferences, and the encouragement provided by a dietitian or nutritionist. As such, the overall response to a dietary intervention reflects both its physiological and biochemical effects and a range of psychological and contextual factors influencing the placebo response. This process underscores the critical need for placebo controls in trials evaluating dietary strategies (Staudacher et al. 2017).

Identifying highly specific and sensitive biomarkers is a key step toward facilitating early diagnosis, which can substantially improve clinical management and patient prognosis. Although many potential biomarkers have been proposed, the supporting evidence has remained preliminary and requires further rigorous validation (Balistreri et al. 2025). Neurotrophic factors are essential in myelination. BDNF, a key neurotrophic factor, is a promising target for the treatment of MS (Albini et al. 2023). Research has shown that MS patients show decreased serum levels of BDNF. Given that the BDNF receptor, TrkB, is abundantly expressed in neurons and astrocytes within MS brain lesions (Stadelmann et al. 2002), the idea of neuroprotective autoimmunity is backed (Cross et al. 2014). BDNF endorses axonal growth, persuades oligodendrocyte progenitor cells, oversees myelination in Schwann cells, and motivates myelin production. The intake of omega‐3 fatty acids raises BDNF levels (Matsuoka et al. 2015; Venna et al. 2009; Wu et al. 2004), which may contribute to improvements in neurological disorders (Reimers and Ljung 2019). Consumption of DHA raises BDNF levels in the hippocampus (Wu et al. 2004, 2008) and increases TrkB receptors, which are involved in signaling pathways related to growth, differentiation, and the division of neural stem cells (Ishii et al. 1993). They increase neurotrophic factors that promote the growth of glial cells in the substantia nigra (Vines et al. 2012). Besides, omega‐3 fatty acids enhance energy metabolism pathways, ultimately increasing BDNF levels (Gomez‐Pinilla 2008). A systematic review and meta‐analysis regarding the effects of omega‐3 fatty acids on BDNF revealed the effectiveness of PUFA supplementation in raising serum BDNF levels in individuals who received the supplements compared to those on a placebo (Ziaei et al. 2024). Additionally, animal research supports the view that BDNF measurements in blood and plasma reflect BDNF levels in brain tissue (Klein et al. 2011). These results convincingly prove the neuroprotective effects of omega‐3 fatty acids, which are mediated by increased BDNF levels in both the brain and serum (Harauma and Moriguchi 2011).

C‐reactive protein (CRP) is a well‐known biomarker of systemic inflammation and an acute‐phase protein produced by hepatocytes in response to inflammatory reactions, such as chronic inflammatory diseases like MS. Hs‐CRP is a more sensitive test for inflammation and may reflect low‐grade systemic inflammation in various psychiatric disorders (Sproston and Ashworth 2018). The impact of omega‐3 fatty acids on serum CRP levels has already been emphasized in research (Fiedler et al. 2005; Saifullah et al. 2007). In individuals with Parkinson disease, n‐3 PUFA intake improves function by reducing inflammatory proteins such as CRP and increasing BDNF gene expression (Reimers and Ljung 2019).

Notably, research on neurodegenerative diseases has shown that combining BDNF and hs‐CRP in a biomarker panel significantly enhances diagnostic and prognostic accuracy compared to using either marker alone. This finding supports their potential utility in facilitating early diagnosis and personalized management of neurodegenerative conditions, including risk assessment and outcome prediction (Balistreri et al. 2025). Additionally, concurrent changes in circulating inflammatory markers and neurotrophic factors may serve as valuable indicators for forecasting cognitive decline in individuals with multiple sclerosis (Talebi et al. 2021).

Although review studies have noted the benefits of omega‐3 fatty acids in MS patients, we need additional studies into the relationship between MS complications and these fatty acids (Tryfonos et al. 2019). Additionally, the impact of omega‐3 fatty acids supplementation on hs‐CRP serum levels has been a topic of debate (Balk et al. 2006; Ebrahimi et al. 2009). Given the absence of research investigating the influence of this nutrient on both BDNF and hs‐CRP serum levels in patients with MS, the current study seeks to examine the effects of n‐3 fatty acid supplementation on serum levels of BDNF and hs‐CRP, as well as on physical activity and chronic fatigue in individuals with MS.

## 2 Materials and Methods

### 2.1 Study Design, Setting, and Subjects

**FIGURE 1:**
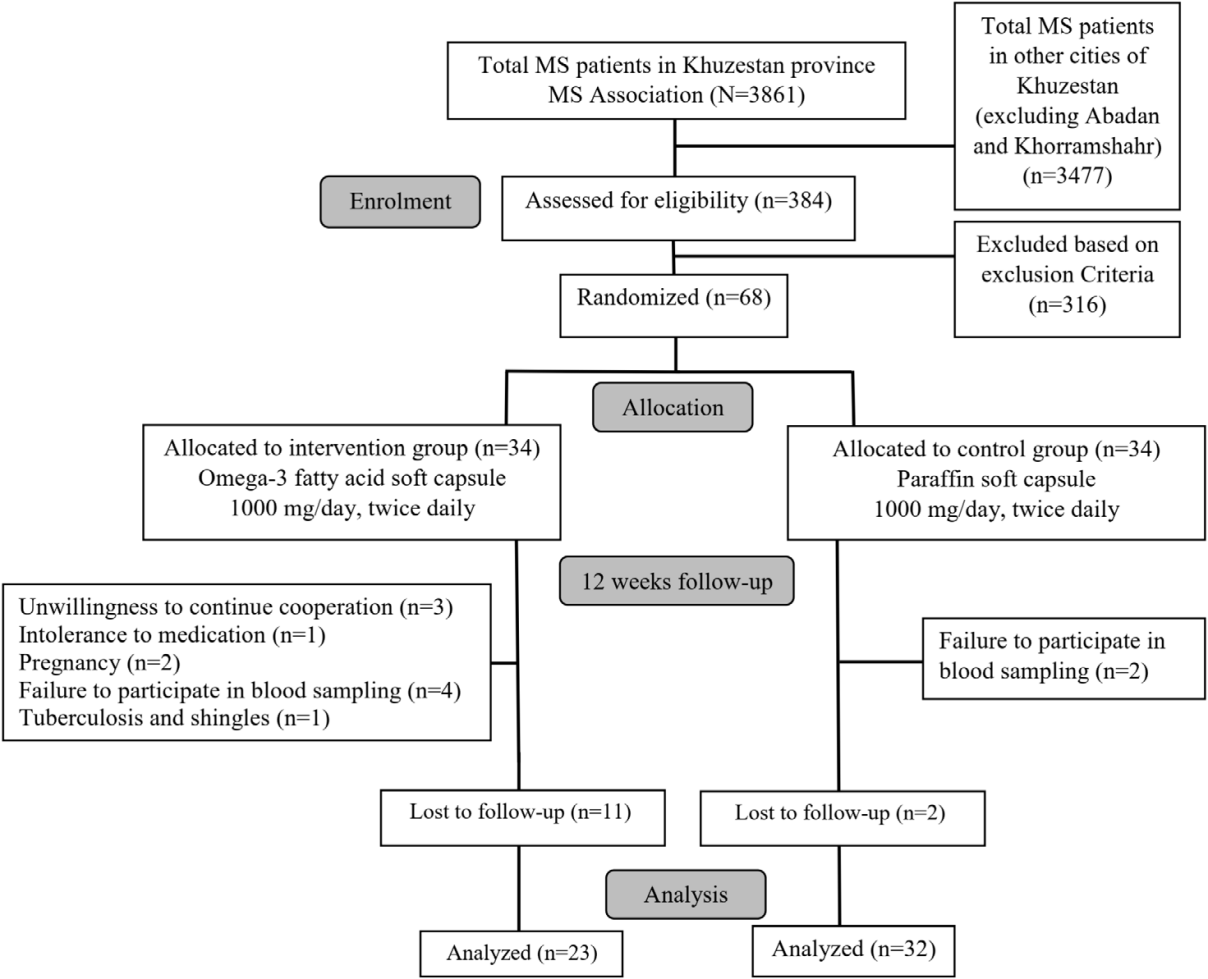
The schematic diagram of study design. The reasons for drop out from analysis are displayed.

A randomized, double‐blind, placebo‐controlled trial was conducted at the School of Medicine, Abadan University of Medical Sciences, Abadan City, Iran, in collaboration with the MS Association of Khuzestan Province from May to August 2024, screening 384 MS patients to include 68 eligible participants aged 16 to 64 ultimately. They were divided into the intervention (*n* = 34) and control (*n* = 34) groups, with 23 and 32 patients, respectively, completing the study for data analysis. Demographic details and medical histories were documented, and patients were assessed for eligibility based on the established inclusion and exclusion criteria (Figure 1).

Patients in the intervention group received n‐3 PUFAs soft capsules (Nextyle, Nexus pharmaceutical company, Lincolnshire, Illinois) with a dose of 1000 mg/d twice a day (a daily dosage of 2000 mg) after a meal for 12 weeks (Ziaei et al. 2024). Each soft gel included fish oil 1000 mg, EPA (eicosapentaenoic acids) 180 mg, and DHA (docosahexaenoic acids) 120 mg.

Patients in the control group received oral liquid paraffin (mineral oil) soft capsules as a placebo with a dose of 1000 mg/day (Zahravai, Tabriz, Iran) twice a day (a daily dosage of 2000 mg) after a meal for 12 weeks.

Patients were assessed over four visits. The first visit, conducted at the start of the first month, involved blood sampling, completion of baseline questionnaires, and distribution of the omega‐3 fatty acids supplement. During the second and third visits—at the beginning of the second and third months, respectively—patients received additional bottles of the supplement. The fourth and final visit, at the end of the third month, included follow‐up blood sampling and completion of post‐intervention questionnaires.

At each visit, patients returned the previous supplement bottle and then received the next month's supplement. If a patient missed any of the four visits after the study was commenced on, they were excluded from the study.

### 2.2 Sample Size Calculation

The sample size was determined based on the study by Hadjighassem et al. (2015) using an assumed effect size of 0.8 for changes in BDNF, with a significance level (*α*) of 0.05 and a statistical power of 80%, yielding a minimum requirement of 11 participants per group. To accommodate potential dropout rates of up to 20%—common in MS trials—and to allow for exploratory analyses of secondary outcomes, such as fatigue and physical activity, we enrolled 34 participants per group. This assumption ensured adequate power for detecting the primary outcome and a sufficient sample size for secondary analyses.

### 2.3 Inclusion and Exclusion Criteria

The research included participants aged between 16 and 64 years who were diagnosed with MS by a neurologist based on McDonald's criteria (Thompson et al. 2018). These individuals had been on a consistent dosage of MS‐modifying medications for no less than 6 months before being enrolled. They had a Kurtzke expanded disability status scale (EDSS) disability score of 5.0 or lower (Torkildsen et al. 2012). Individuals were excluded from participation if they were involved in any other clinical trials, experienced a recurrence, or received corticosteroid treatment within the last month, were simultaneously treated with antibiotics and antihypertensive medications, had acute or severe conditions besides MS, were pregnant, had a current or past history of ventricular arrhythmia, or had general health issues that could hinder their participation.

### 2.4 Randomization Description

In this study, a simple randomization method was applied to create a random sequence using a Table of random numbers. The researcher first predetermined the order of reading the numbers in the Table (from above) and then considered even numbers. These data emphasize the importance of considering the “placebo effect” when evaluating the impact of experimental supplements on the natural course of MS.

As a default for the intervention (A) and odd numbers for the control group (B), she then placed her hand on one of the numbers, moved in one of the predetermined directions, recorded the numbers, and assigned them to different groups (Suresh 2011).

### 2.5 Allocation Concealment

In this study, opaque sealed and waxed packets were used sequentially to hide random allocation (numbered, sealed, opaque packets). In this method, after creating a random sequence based on the research sample size, several packets with aluminum wrapping (to obscure the contents of the packets) were prepared. The created random sequences were then documented on a card, and the cards were placed in the letter packets to preserve the random sequence. The outside of the packets was numbered in a similar order. Finally, the packets were sealed and located inside a container. The packets were opened in order, and the assigned group of participants was revealed. An independent medical student (researcher) prepared placebo bottles with the same color and shape as n‐3 supplement bottles and put them in the envelope according to the allocation order. These supplements could not be distinguished because they were packaged in cans with the same color, shape, and label. In the third step, implementing the random allocation process, one of the researchers (a faculty member) created a random sequence, a student who was aware of the study examined the participants in terms of inclusion and exclusion criteria, enrolled them in the study, and another medical student allocated the patients into the study groups. The researcher who created the random sequence must be separate from other researchers in the stage of enrollment or allocation to reduce possible bias (Miola et al. 2024).

### 2.6 Blinding

This investigation was carried out using a double‐blind approach. Accordingly, neither the patients nor the researchers knew which study groups the participants had been assigned to. The placebo soft gels were made to closely resemble the n‐3 supplement soft gels regarding color, odor, bottle appearance, and administration method, allowing for effective blinding for both study participants and researchers (Gori et al. 2025).

### 2.7 Baseline Demographic, Anthropometric, and Clinical Variables

To obtain demographic and clinical information, including age, sex, educational levels, EDSS scores, and body mass index (BMI) of the patients, data from medical records registered at the MS Association of Khuzestan Province were utilized. The categories of age and EDSS were derived from Patti et al. study (2021) with the following classification: age < 40 and ≥ 40 years, EDSS < 3 and ≥ 3, respectively. For binary classification, BMI was categorized as < 25 and ≥ 25 kg/m^2^. Education level was classified into three categories from under diploma (primary/secondary school) to academic (bachelor's/master's/doctoral degree) (Lefort et al. 2025).

### 2.8 Dietary Intake Assessment of n‐3 Polyunsaturated Fatty Acids Sources

Dietary intake of n‐3 PUFAs sources and supplement use information was collected at the initial screening visit by a validated, adapted Persian version of the food frequency questionnaire (FFQ), developed by Abedimanesh et al. (2023). This tool aims to assess the dietary intake of all sources of n‐3 fatty acids, including any oral supplements, among a healthy Iranian population by relating dietary data to n‐3 PUFA levels found in red blood cells and utilizing a 3‐day food record. The FFQ was a brief and user‐friendly self‐administered questionnaire that included 14 items, featuring 12 food items high in n‐3 PUFAs, according to Iranian eating habits, in addition to two questions regarding fish oil and omega‐3 fatty acids supplements.

Given the significant intake of red meat, poultry, and processed meat as key sources of n‐3 PUFAs in the Iranian diet, they were included in the FFQ. Although omega‐3 fatty acids‐enriched eggs are not commonly consumed in Iran, they were also added to the questionnaire. Portion sizes for each food listed were based on the most frequently eaten quantities within the general Iranian community. To clarify portion sizes and consumption frequencies for participants, a 30‐min training session was led by a qualified nutritionist at the study location. During this training, portion sizes were illustrated using visual aids or household measurement tools. Participants with MS were asked to report their intake of fish, shrimp, and other seafood, along with various types of meat (red, processed, and white) in grams on a daily, weekly, and monthly basis. The intake of canned tuna, omega‐3 fatty acids‐enriched eggs, and walnuts was recorded as quantities, while the use of canola oil, flaxseed, and flaxseed oil was indicated in teaspoons. n‐3 PUFA supplements were characterized as n‐3 fatty acids, PUFA, DHA, EPA, ALA, or cod liver oil capsules, with quantities or tablespoons for liquid forms noted in the FFQ. Information regarding self‐reported n‐3 PUFA supplementation was gathered during the initial screening visit with the participants.

### 2.9 Primary Outcomes

#### 2.9.1 Measurement of BDNF

Blood samples were collected from all participants in a fasting manner at the study's start and end to assess serum BDNF levels, with samples drawn between 9:00 AM and 12:30 PM, clotted for an hour at room temperature, and then centrifuged at 2000 rpm for 10 min at 4°C and stored at −80°C in 0.2 mL tube strips (Sarstedt, Multiply μStripPro) before analysis using ELISA kits (Padgin Teb Co., Tehran, Iran), resulting in intra‐ and interassay CVs of less than 10% and less than 12%, respectively, for BDNF concentrations between 0.4 and 12.8 ng/mL, with a sensitivity of 0.05 ng/mL.

#### 2.9.2 Measurement of hs‐CRP

The hs‐CRP serum levels were measured using a sandwich immunodetection method. Ichroma hsCRP kit (Document No.: INS‐CH‐EN), a fluorescence immunoassay, was used for the quantitative determination of hs‐CRP in the serum of patients. The ichroma test instrument automatically calculated the test results and presented the hs‐CRP concentration of the sample in mg/L. The operational range was between 0.1 and 10 mg/L. For more analysis, the amount of hs‐CRP serum levels of the patients was classified into three ranges as follows: < 1, 1–3, and > 3 mg/L as low‐, intermediate‐, and high‐risk groups for global CVD, respectively, based on data obtained from population‐based studies, the AHA/CDC (American Heart Association/Centers for Disease Control) working group on markers of inflammation in CVD (Roberts et al. 2004).

### 2.10 Secondary Outcomes

#### 2.10.1 Physical Activity

A short version of the International Physical Activity Questionnaire (IPAQ) was used to assess physical activity (PA) at the start of the study and again 12 weeks after the intervention, with results interpreted following the method outlined in Mynarski et al. study (2012).

#### 2.10.2 Modified Fatigue Impact Scale (MFIS)

At the beginning of the study and 12 weeks after the start of the intervention, chronic fatigue scale score was obtained by MFIS questionnaire consisting of 10 “physical” items, 10 “cognitive” items, and 20 “social” items to measure the extent of chronic fatigue of patients in the last 4 weeks (Cordano et al. 2025). All patients completed MFIS questionnaires during warm seasons at Abadan City, Khuzestan Province, Iran, which is an area with large seasonal differences in outdoor temperature.

### 2.11 Safety

Safety was evaluated by adverse events reported by MS patients, vital signs, and physical and neurological examinations.

### 2.12 Compliance

Compliance involved the regular intake of n‐3 PUFA supplements and/or soft capsules containing a placebo. At the three‐month mark, compliance was evaluated through a count of the capsules and tracked via a diary, which was examined during in‐person visits at the one, two, and three‐month intervals. Taking more than 80% of prescribed supplements or placebo was considered a desirable level of compliance. To increase participant adherence, patients were instructed on the correct way to take the supplement or placebo and reminded regularly by text messages and phone calls.

### 2.13 Statistical Analysis

The Kolmogorov–Smirnov test was used to assess the normality of outcome measures. As the data were not normally distributed (*p* < 0.05), non‐parametric tests were used. Between‐group differences were analyzed using the Mann–Whitney *U* test, while within‐group changes were assessed using the Wilcoxon signed‐rank test. Quade's analysis of covariance (ANCOVA) was used to compare group differences in BDNF and hs‐CRP levels, adjusting for baseline values and relevant covariates (BMI, poultry consumption, and sex).

We performed a per‐protocol analysis, including only participants who completed the 12‐week intervention and all required visits (*n* = 23 in the intervention group, *n* = 32 in the control group), as complete pre‐ and post‐intervention biomarker data were essential for the analysis.

Independent sample *t* test and Chi‐square test were performed to evaluate the demographic, anthropometric, clinical, levels of fatigue, physical activity, MS types, and sources of n‐3 dietary intake among the study groups. The Mann–Whitney *U* test was used to assess BDNF and hs‐CRP serum levels that were not normally distributed. The Wilcoxon signed‐rank test was used for comparisons within groups, and Quade's rank ANCOVA was chosen to analyze group differences in BDNF and hs‐CRP levels due to persistent non‐normality of the data, even after attempted transformations (e.g., logarithmic). This method adjusts for baseline values and covariates (BMI and poultry consumption) using rank‐transformed data, ensuring robust analysis without violating parametric assumptions (Vickers 2005). Adjustments for BMI and poultry consumption were made in Quade's ANCOVA to account for significant baseline differences between groups (*p* = 0.028 and *p* = 0.035, respectively), as BMI influences inflammatory markers (Arcari et al. 2008) and poultry is a dietary source of omega‐3 fatty acids (Cartoni Mancinelli et al. 2022).

Linear regression analysis was conducted to examine the influence of study variables on changes in serum BDNF and hs‐CRP levels from pre to post‐intervention. Additionally, the Chi‐square test was used to compare categorical distributions of hs‐CRP levels between groups at both time points. All statistical analyses were performed using IBM SPSS Statistics, version 26.0 (IBM Corp., Armonk, NY).

To account for the baseline imbalance in hs‐CRP risk categories (*p* = 0.012), Quade's ANCOVA was adjusted for preintervention hs‐CRP values, BMI, poultry consumption, and sex, the latter showing a significant association with changes in hs‐CRP (*p* = 0.005).

## 3 Results

A total of 68 patients with MS took part in the study, with 34 assigned to the intervention and 34 to the control group. After 12 weeks, 11 individuals from the intervention and 2 from the control group were lost to follow up. In the control group, two participants dropped out due to nonparticipation in blood sampling. In the intervention group, there were 11 dropouts: three due to withdrawal of consent, one due to intolerance to the supplementation, two due to pregnancy, four due to failure to participate in blood sampling, and one due to a diagnosis of tuberculosis and shingles. However, no dropouts were seen due to noncompliance and gastrointestinal effects (nausea and indigestion) in the present study (Figure 1).

A sensitivity analysis was conducted to compare baseline characteristics between completers (*n* = 55) and dropouts (*n* = 13). No significant differences were found in age, sex, BMI, EDSS, or dietary intake (*p* > 0.05), suggesting that dropout‐related selection bias was minimal.

**FIGURE 2:**
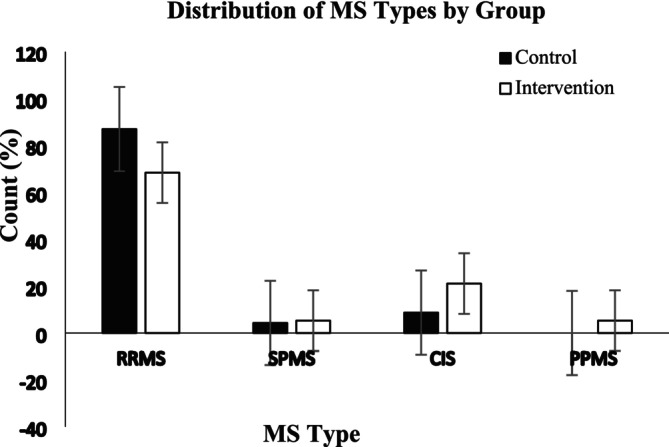
The bar chart illustrates the frequency distribution of various MS types among study groups, revealing no significant differences (*p* = 0.424) after chi‐squared test analysis, with MS types defined as RRMS (relapsing–remitting), SPMS (secondary progressive), CIS (clinically isolated syndrome), and PPMS (primary progressive).

Finally, 23 patients in the intervention group and 32 in the control group were retained for conclusive analysis. They completed the study, where baseline analysis revealed no significant differences apart from weight and BMI (*p* = 0.043 and *p* = 0.028, respectively) (Table 1), and the distribution of MS types was consistent across groups (*p* = 0.531), with RRMS being the most common type (Figure 2).

**TABLE 1:** Baseline demographic, anthropometric, clinical, chronic fatigue related situation, and physical activity levels of MS patients.

Variables	Group		*p*
	Intervention (*n* = 34)	Control (*n* = 34)	
Male (%)	6 (17.6)	11 (32.4)	0.161^a^
Age (year)	36.6 ± 10.1	40.6 ± 8.6	0.085^b^
Age category			
< 40	21 (61.8%)	13 (39.4%)	0.056^a^
≥ 40	13 (38.2%)	20 (60.6%)	
Educational levels			
Under diploma	11 (32.4)	8 (23.5)	0.078^a^
Diploma	9 (26.5)	18 (52.9)	
Academic	14 (41.2)	8 (23.5)	
Weight (kg)	65.8 ± 16.3	73.2 ± 13.3	0.043^b^
BMI (kg/m^2^)	24.4 ± 4.8	27 ± 4.7	0.028^b^
BMI category			
< 25	17 (50)	12 (35.3)	0.163^a^
≥ 25	17 (50)	22 (64.7)	
EDSS	1.8 ± 1.9	1.6 ± 1.0	0.567^b^
EDSS category			
< 3	13 (65)	19 (82.6)	0.166^a^
≥ 3	7 (35)	4 (17.4)	

Abbreviations: BMI, body mass index; EDSS, expanded disability status scale.

^a^ The data analysis utilized a chi‐squared test for categorical data (presented as number and percentage).

^b^ Study groups indicate a significant (*p* < 0.05) difference in weight (Kg) and BMI (Kg/m^2^) (Independent *t*‐test was applied for continuous data presented as mean ± standard deviation).

### 3.1 Physical Activity Levels and Chronic Fatigue‐Related Situation of MS Patients Before and After the Intervention

Based on the IPAQ, average levels of weekly physical activity—including high intensity, moderate intensity, and total activity measured in MET (metabolic equivalent of task)—were lower in the intervention group compared to the control group. However, the differences were not statistically significant (*p* > 0.05). Similarly, no significant differences were observed in total MFIS scores or in its physical, cognitive, and psychosocial subscales at baseline or following the intervention.

Additionally, at baseline, 97.1% of participants in the intervention group and 88.2% in the control group had low levels of daily physical activity (≤ 2182 MET‐min/week), with no significant difference between groups (*p* > 0.05). After the 12‐week follow‐up, 21.7% of participants in the intervention group and 9.7% in the control group exhibited high levels of physical activity (> 2182 MET‐min/week). However, this difference was also not statistically significant (*p* > 0.05) (Table 2).

**TABLE 2:** The comparison of physical activity levels and chronic fatigue‐related situations of MS patients before and after the intervention.

Variables	Before intervention			After intervention		
	Intervention (*n* = 34)	Control (*n* = 34)	*p*	Intervention (*n* = 23)	Control (*n* = 32)	*p*
Physical subscale in MFIS^a^	12.0 ± 7.6	14.7 ± 7.9	0.173^b^	10.04 ± 7.6	13.6 ± 9.5	0.153^b^
Cognitive subscale in MFIS	12.5 ± 7.5	15 ± 8.0	0.195^b^	10.2 ± 8.2	13.9 ± 10.2	0.16^b^
Psychosocial subscale in MFIS	3.1 ± 2.1	4 ± 1.9	0.074^b^	2.5 ± 2.2	3.7 ± 2.4	0.068^b^
Total MFIS	27.7 ± 16.2	33.6 ± 16.7	0.142^b^	22.7 ± 17.2	31.5 ± 21.9	0.123^b^
PA1^c^ (MET min/week)	65.1 ± 180	110.6 ± 262.7	0.408^b^	60 ± 136.6	56.9 ± 156.8	0.941^b^
PA2^d^ (MET min/week)	190.9 ± 417.8	565.7 ± 1089.2	0.065^b^	701.7 ± 938.7	645.5 ± 1029.1	0.939^b^
PA3^e^ (MET min/week)	162.2 ± 389.7	117.4 ± 204.8	0.555^b^	188.3 ± 445.6	85 ± 126.1	0.838^b^
DPA^f^ (MET min/week)	418.2	793.7	0.109^b^	948.7 ± 1065.5	787.4 ± 1011.4	0.574^b^
High DPA^g^ (> 2182 MET min/week)	1 (2.9)	4 (11.8)	0.163^g^	5 (21.7)	3 (9.7)	0.198^g^
Low DPA^g^ (≤ 2182 MET min/week)	33 (97.1)	30 (88.2)		18 (78.3)	28 (90.3)	

^a^ Modified Fatigue Impact Scale.

^b^ Independent *t*‐test was applied for continuous data (presented as mean ± standard deviation).

^c^ Weekly physical activity at high intensity.

^d^ Weekly physical activity at moderate intensity.

^e^ Weekly physical activity at low intensity.

^f^ Declared weekly physical activity.

^g^ The data analysis utilized a chi‐squared test for categorical data (presented as number and percentage).

### 3.2 Dietary Intake Measurement of n‐3 Fatty Acids

To evaluate the impact of dietary n‐3 PUFA intake on the results, FFQ questionnaires were distributed among the patients before the intervention, indicating significant differences in poultry meat consumption between the control and intervention groups (*p* = 0.035). At the same time, other sources of n‐3 fatty acids did not display any significant differences, and no participants in either group were using any n‐3 PUFA supplements before the start of the intervention. Additionally, the participants had no dietary restrictions and consumed their routine diet (Table 3).

**TABLE 3:** Sources of omega‐3 fatty acids intake in patients with multiple sclerosis.

Variables	Group		*p* ^a^
	Intervention (*n* = 34)	Control (*n* = 34)	
Fish (g/day)	12.8 ± 11.2	10.6 ± 8.5	0.373
Shrimp (g/day)	5.3 ± 8.9	3.8 ± 8.8	0.511
Tuna (g/day)	2.3 ± 5.02	2.1 ± 5.3	0.894
Red meat (lamb, mutton, beef, and weal) (g/day)	21 ± 17.7	21 ± 31	0.903
Processed meats (sausages, hot dogs, hamburgers) (g/day)	12 ± 23.6	5.8 ± 11.7	0.180
Poultry meat (g/day)	19.4 ± 15.9	35.5 ± 39.8	0.035
Eggs (g/day)	33.6 ± 32.8	35 ± 35.4	0.871
Walnuts (g/day)	4.9 ± 5	4.2 ± 6.0	0.590
Canola Oil (g/day)	0 ± 0	0.2 ± 1.0	0.321
Flaxseed (g/day)	0.2 ± 0.8	0 ± 0	0.288

^a^ Independent *t*‐test was applied to analyze data. Data have been presented as mean ± standard deviation.

### 3.3 BDNF Serum Levels

Table 4 presents the comparison of baseline BDNF serum levels between the intervention and control groups, revealing no significant differences (*p* = 0.321). At the same time, omega‐3 fatty acids supplementation led to a significant increase in BDNF in the intervention group (*p* < 0.001) and a similar increase in the control group (*p* < 0.001). After the intervention, however, the Mann–Whitney *U* test indicated no significant difference between groups (*p* = 0.427). Also, the Quade non‐parametric ANCOVA, including the pre‐intervention values of the BDNF serum levels and dietary intake of poultry meat and BMI (as covariates to adjust for preexisting differences), bolstered that omega‐3 fatty acids supplementation had no influence on BDNF levels (*p* = 0.644).

**TABLE 4:** The within‐ and between‐group comparison of BDNF serum levels (ng/mL) before and after the intervention.

BDNF (ng/mL)	Time		*p*	*p*
Group	Before	After		
Intervention (*n* = 23)	2.15 ± 1.11	4.34 ± 1.37	< 0.001^a^	0.644^b^
Control (*n* = 32)	2.45 ± 1.16	4.58 ± 1.63	< 0.001^a^	
*p*	0.321^c^	0.427^c^		

^a^ Omega‐3 fatty acids supplementation increased significantly BDNF serum levels in the intervention and control group (*p* < 0.001). The *p* value was derived from the Wilcoxon signed‐rank test.

^b^ The *p* value was obtained from the Quade nonparametric ANCOVA test.

^c^ The *p* value was derived from the Mann–Whitney *U* test.

**FIGURE 3:**
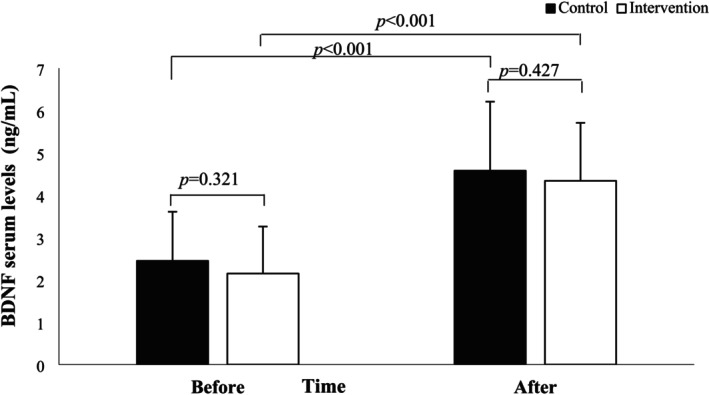
Within‐group and between‐group comparison of BDNF serum levels (ng/mL) of MS patients before and after the omega‐3 fatty acids supplementation and placebo administration. There was no significant difference before and after 12 weeks of follow‐up between the intervention and control groups.

Figure 3 illustrates the within‐ and between‐group comparison of BDNF serum levels in MS patients pre‐ and post‐omega‐3 fatty acids supplementation and placebo, showing no significant differences through the Mann–Whitney *U* test after 12 weeks. However, both groups indicated a significant increase in BDNF levels compared to baseline (*p* < 0.001).

The effects of omega‐3 fatty acid supplementation, demographic, anthropometric, and clinical characteristics, as well as chronic fatigue‐related situations and physical activity levels, on BDNF serum levels in MS patients were evaluated using simple linear regression analysis. The results showed that the omega‐3 fatty acid supplementation, sex, age, education, BMI, EDSS, chronic fatigue subscales, and physical activity levels did not affect the BDNF serum level changes (Table 5).

**TABLE 5:** Simple linear regression of BDNF serum levels (ng/mL) with study parameters.

Model	Univariate regression		
	B	SE	*p*
Group			
Control	Ref	—	—
Intervention	0.100	0.419	0.812
Sex			
Male	Ref	—	—
Female	0.616	0.486	0.211
Age (year)			
≤ 40	Ref	—	—
> 40	−0.260	0.412	0.531
Educational levels			
Under diploma	Ref	—	—
Diploma	0.181	0.510	0.725
Academic	−0.459	0.539	0.398
BMI (kg/m^2^)			
< 25	Ref	—	—
≥ 25	0.223	0.414	0.592
EDSS			
< 3	Ref	—	—
≥ 3	0.189	0.548	0.732
Physical subscale in MFIS	0.028	0.027	0.308
Cognitive subscale in MFIS	0.038	0.026	0.153
Psychosocial subscale in MFIS	−0.028	0.099	0.780
PA1^a^ (MET min/week)	0.000	0.001	0.840
PA2^b^ (MET min/week)	0.000	0.000	0.063
PA3^c^ (MET min/week)	−0.001	0.001	0.114
DPA^d^ (MET min/week)	0.000	0.000	0.240

*Note:* Dependent Variable: BDNF change before and after the intervention.

Abbreviations: BMI, body mass index; EDSS, Expanded Disability Status Scale; MFIS, Modified Fatigue Impact Scale.

^a^ Weekly physical activity at high intensity.

^b^ Weekly physical activity at moderate intensity.

^c^ Weekly physical activity at low intensity.

^d^ Declared weekly physical activity.

### 3.4 hs‐CRP Serum Levels

Table 6 compares the within‐group and between‐group hs‐CRP serum levels. The Mann–Whitney *U* test indicated that the intervention and control groups showed no significant differences in hs‐CRP serum levels when comparing measurements taken before and after the intervention (*p* = 0.996, *p* = 0.695, respectively). The within‐group comparison of hs‐CRP serum levels before and after the intervention with the Wilcoxon signed‐rank test showed that the consumption of 1000 mg/d twice a day of omega‐3 fatty acids supplementation and the oral paraffin soft capsules as a placebo with a dose of 1000 mg/d twice a day for 12 weeks led to no significant change in hs‐CRP serum levels in the intervention (*p* = 0.948) and control (*p* = 0.975) groups, respectively.

**TABLE 6:** The within‐ and between‐group comparison of hs‐CRP serum levels (mg/L) before and after the intervention.

hs‐CRP (mg/L)	Time		*p*	*p*
Group	Before	After		
Intervention (*n* = 23)	2.45 ± 3.15	2.27 ± 2.59	0.948^a^	0.533^b^
Control (*n* = 32)	2.45 ± 2.49	2.53 ± 2.40	0.975^a^	
*p*	0.996^c^	0.695^c^		

^a^ The *p* value was calculated using the Wilcoxon signed‐rank test.

^b^ The *p* value was determined via the Quade non‐parametric ANCOVA test.

^c^ The *p* value was obtained through the Mann–Whitney *U* test.

Furthermore, the findings from the Quade nonparametric ANCOVA, which was adjusted for preintervention values and dietary intake of poultry meat and BMI as covariates, revealed that omega‐3 fatty acids supplementation had no impact on hs‐CRP serum levels. Also, there was no significant difference between the two groups concerning this measure (*p* = 0.533).

**FIGURE 4:**
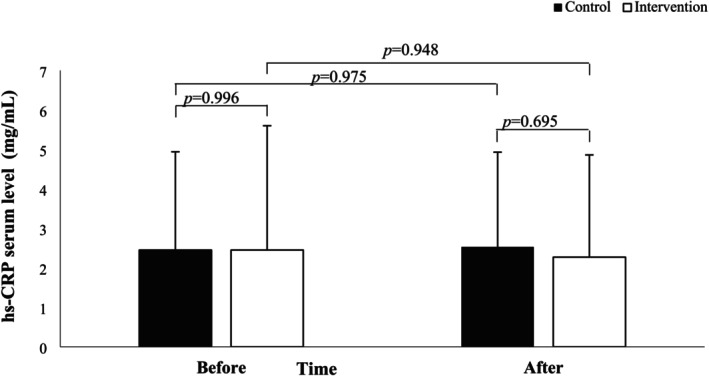
The comparison of hs‐CRP serum levels (mg/L) in MS patients before and after 12 weeks of omega‐3 fatty acids supplementation and placebo showed no significant difference between the intervention and control groups.

Figure 3 illustrates the within‐ and between‐group comparison of hs‐CRP serum levels (mg/L) of MS patients before and after the omega‐3 fatty acids supplementation and placebo administration. The Mann–Whitney *U* test showed no significant difference between the intervention and control groups before and following the 12‐week follow‐up (Figure 4).

The impact of omega‐3 fatty acids supplementation, demographic, anthropometric, clinical characteristics, chronic fatigue‐related situation, and physical activity levels variables of MS patients on the changes in hs‐CRP serum levels was evaluated using the simple linear regression analysis (Table 7). The results indicated that the omega‐3 fatty acids supplementation, age, education, BMI, EDSS, chronic fatigue subscales, and physical activity levels did not affect the changes in hs‐CRP serum levels. However, the hs‐CRP serum level changes were different in both sexes as the mean of hs‐CRP serum level changes was significantly 1.5 times higher in female MS patients than in male ones (*p* = 0.005).

**TABLE 7:** Simple linear regression of hs‐CRP serum levels (mg/L) changes.

Variable	Univariate regression		
	*B*	SE	*p*
Group			
Placebo	Ref	—	—
Omega‐3 fatty acids	−0.238	0.450	0.599
Sex			
Male	Ref	—	—
Female	1.472	0.496	0.005
Age (year)			
≤ 40	Ref	—	—
> 40	−0.005	0.446	0.991
Educational levels			
Under diploma	Ref	—	—
Diploma	0.541	0.554	0.333
Academic	0.275	0.573	0.634
BMI (kg/m^2^)			
< 25	Ref	—	—
≥ 25	0.083	0.451	0.854
EDSS			
< 3	Ref	—	—
≥ 3	−0.205	0.605	0.736
Physical subscale in MFIS	−0.023	0.029	0.430
Cognitive subscale in MFIS	−0.003	0.028	0.904
Psychosocial subscale in MFIS	−0.079	0.104	0.451
PA1^a^ (MET min/Week)	0.000	0.001	0.830
PA2^b^ (MET min/Week)	0.000	0.000	0.606
PA3^c^ (MET min/Week)	0.001	0.001	0.392
DPA^d^ (MET min/Week)	0.000	0.000	0.812

*Note:* Dependent variable: hs‐CRP change before and after the intervention.

Abbreviations: BMI, body mass index; EDSS, Expanded Disability Status Scale; MFIS, Modified Fatigue Impact Scale.

^a^ Weekly physical activity of high intensity.

^b^ Weekly physical activity of moderate intensity.

^c^ Weekly physical activity of low intensity.

^d^ Declared weekly physical activity.

Table 8 demonstrates the comparison of hs‐CRP serum levels between the intervention and control groups, both before and following the intervention. According to the findings from the chi‐squared test, a statistically significant difference existed between the groups before the intervention (*p* = 0.012). Thus, before the intervention, a larger percentage of participants in the intervention group were placed in the low‐risk category (55.9%) in contrast to the control group (29.4%). The number of individuals classified as high‐risk (> 3 mg/L) was marginally greater in the intervention group (29.4%) than in the control group (23.5%). Following the n‐3 PUFA intervention, there was a significant decline in the proportion of participants categorized as low‐risk (from 55.9% to 43.5%), while the intermediate‐risk category showed an increase (from 14.7% to 43.5%). The percentage of those in the high‐risk group (> 3 mg/L) significantly decreased from 29.4% to 13% (*p* = 0.01).

**TABLE 8:** The comparison of the range of hs‐CRP serum levels (mg/L) between and within study groups before and after the intervention.

hs‐CRP (mg/L)	Time		*p* ^a^
Group	Before (*n* = 34 in both groups)	After (*n* = 23 in intervention and *n* = 32 in control groups)	
Intervention			
< 1^c^	19 (55.9)	10 (43.5)	
1–3^d^	5 (14.7)	10 (43.5)	0.01
> 3^e^	10 (29.4)	3 (13)	
Control			
< 1^c^	10 (29.4)	7 (22.6)	
1–3^d^	16 (47.1)	16 (51.6)	0.001
> 3^e^	8 (23.5)	8 (25.8)	
*p*	**0.012** ^**b**^	0.216	

^a^ The percentage of patients in the high‐risk group (> 3 mg/L) significantly decreased (*p* < 0.05). The within‐group analysis also revealed a significant change (*p* < 0.05). Within‐group comparison was applied by chi‐squared test.

^b^ A statistically significant difference is indicated between the study groups before the intervention (*p <* 0.05). Between‐group comparison was applied by chi‐squared test. Data were presented as number (percent).

^c^ Low risk in cardiovascular disease.

^d^ Intermediate risk in cardiovascular diseases.

^e^ High risk in cardiovascular disease.

The control group experienced minimal changes, with the distribution of risk categories remaining relatively stable. The within‐group analysis also revealed a significant change (*p* = 0.001). Following the intervention, the difference between the intervention and control groups was not statistically significant (*p* = 0.216). This analysis suggested that the intervention facilitated a transition from high to intermediate risk within the intervention group. Nevertheless, after the n‐3 PUFA intervention, there was no significant difference between the two groups, indicating that the control group also experienced some level of change. While the intervention seemed to lower hs‐CRP levels in the high‐risk category, it did not substantially outperform the control group in terms of overall risk distribution.

### 3.5 Safety and Compliance

n‐3 PUFAs in the form of soft capsules were well tolerated, and there was no serious adverse event. However, one patient in the intervention group reported dyspepsia and nausea, and patients in the control group reported stomach pain (*n* = 1), increased menstrual bleeding (*n* = 1), headache (*n* = 2), bone dryness (*n* = 1), leg pain (*n* = 1), and polyuria (*n* = 1). A significant point in our study was that patients in the control group reported improved quality of life (*n* = 1) and decreased leg jerking during sleep (*n* = 1). Moreover, compliance by pill count was > 90% for the intervention and > 80% for the control groups. Nonetheless, there was no significant difference in the mean and standard deviation of compliance between the two study groups throughout the three months of follow‐up (Table 9).

**TABLE 9:** Mean and standard deviation of compliance during the 3‐month follow‐up.

Time	Intervention	Control	*p* ^a^
First month of follow‐up (*n* = 31 for both groups)	92.3 ± 12.8	82.7 ± 21.3	0.071
Second month of follow‐up (Intervention: *n* = 26, Control: *n* = 29)	92.7 ± 14.8	86.3 ± 21.5	0.415
Third month of follow‐up (Intervention: *n* = 25, control: *n* = 28)	94.9 ± 7.7	85.4 ± 21.9	0.267
Total compliance	94.2 ± 6.8	84.7 ± 18.8	0.062

^a^ The *p* value was derived from the Mann–Whitney *U* test. Data were presented as mean ± SD.

## 4 Discussion

Our findings revealed that omega‐3 fatty acids supplementation as an augmentation therapy to improve BDNF and hs‐CRP serum levels, physical activity, and chronic fatigue in MS was not significantly different from placebo in this randomized, double‐blind, placebo‐controlled trial. However, n‐3 PUFA supplementation at the dose given was well tolerated over 3 months. In this study, we evaluated the effect of n‐3 PUFAs soft capsules on a panel of molecules, including BDNF and systemic inflammatory index, hs‐CRP, in MS patients.

### 4.1 BDNF Serum Levels and n‐3 Supplements in MS

The results of our research demonstrated that a daily intake of 1000 mg of omega‐3 fatty acids, taken twice a day for 12 weeks, led to a significant rise in BDNF serum levels in the intervention group compared to baseline measurements. At the same time, the use of oral paraffin soft capsules as a placebo, administered at a dose of 1000 mg per day twice daily for 12 weeks, also showed an increase in BDNF serum levels from baseline. However, omega‐3 fatty acids supplementation did not lead to any changes in BDNF serum levels, and there was no significant difference observed between the intervention and control groups before and after the 12‐week follow‐up period.

The results also showed that the omega‐3 fatty acids supplementation, sex, age, education, BMI, EDSS, chronic fatigue subscales, and physical activity levels did not affect the changes in BDNF serum levels.

A review of the database did not reveal any research focused on the impact of n‐3 supplements on serum BDNF levels in individuals with MS. The findings from this study indicated no positive effects of omega‐3 fatty acids supplementation on BDNF serum levels. Nevertheless, a systematic review and meta‐analysis conducted in 2024 demonstrated that omega‐3 fatty acid supplements were linked to a noteworthy increase in serum BDNF levels among the general population taking the supplementscompared to the placebo group (Ziaei et al. 2024). These results suggest that n‐3 may have a positive effect on BDNF levels in healthy adults, but further research is needed to confirm this effect in patients with MS. Besides, the findings of two systematic reviews and meta‐regression analyses investigating the impact of n‐3 supplementation on BDNF showed that the effects were larger when supplements were used for longer than 10 weeks, doses under 1500 mg/d, and adults under 50 years of age (Liu et al. 2023; Sohouli et al. 2023). Considering the dose of 2000 mg/d used in the present study, it is probable that administration of such a high dose of n‐3 supplements in patients with MS could lead to ineffectiveness on BDNF serum levels. This outcome should be explored in more detail in subsequent studies.

Three key sources of ambiguity may arise in this context. Notably, a significant increase in serum BDNF levels among participants receiving liquid paraffin as a placebo raises concerns about its potential confounding effects on the study outcomes. Ideally, a placebo should be chemically inert; however, many omega‐3 fatty acid intervention trials have used oils that may possess some biological activity. The saturated, monounsaturated, and omega‐6 polyunsaturated fatty acids—found in varying proportions in oils like olive, corn, safflower, sunflower, and coconut—can affect the inflammatory pathways. When such biologically active oils are used as comparators, they may either obscure or enhance the observed effects of omega‐3 fatty acid interventions, depending on the choice of placebo (Olshansky et al. 2020). Therefore, using mineral oil as a placebo helped eliminate the potential confounding influence of omega‐6 fatty acids in the control group (Paixao et al. 2017). Conversely, formal assessment—such as those presented during an FDA Advisory Committee meeting—suggested that mineral oil had minimal to no impact on hs‐CRP levels when used as the placebo (Bhatt et al. 2020).

Inconsistent changes in other inflammatory markers have been reported across various pathological conditions in which mineral oil was used as a placebo. These markers include interleukin (IL)‐6 (Paixao et al. 2017; Rashidmayvan et al. 2019), IL‐1β (Lotfi‐Dizaji et al. 2019; Paixao et al. 2017; Rashidmayvan et al. 2019; Tan et al. 2018), IL‐10 (Gharekhani et al. 2014), tumor necrosis factor‐α (Rashidmayvan et al. 2019), intercellular adhesion molecule 1 (Bays et al. 2013), and monocyte chemoattractant protein‐1 (Lotfi‐Dizaji et al. 2019). However, these variations have generally been minor. Due to their inert properties, highly refined, pharmaceutical‐grade mineral oils are commonly used in clinical trials, especially those investigating oil‐based formulations, such as omega‐3 fatty acids. In 2015, the European Commission revised its regulations to classify pharmaceutical‐grade mineral oils as substances/active ingredients posing no health risk (Olshansky et al. 2020). In the current trial, the placebo consisted of a purified blend of saturated hydrocarbons with a defined chemical structure. To explore possible mineral oil safety and effects, we conducted a literature search to identify studies employing mineral oil placebos.

Adverse events linked to mineral oil were primarily gastrointestinal and aligned with its known effects as a lubricant laxative. Reported changes in serum lipoprotein levels, hs‐CRP, and other biomarkers were inconsistent and generally neither statistically nor clinically significant. Current evidence indicates that paraffin oil (2–4 g/d) does not consistently interfere with the absorption of essential nutrients. Furthermore, capsules containing liquid paraffin appear to have no significant impact on the absorption or efficacy of medications, nor related clinical outcomes. Therefore, when used as a placebo in clinical trials at these quantities, mineral oil is unlikely to influence study results or alter overall conclusions (Olshansky et al. 2020). Previous research on polyunsaturated fatty acid supplementation primarily used olive oil as a placebo (Paty 1983; Weinstock‐Guttman et al. 2005), which may suppress nuclear factor β activation (Brunelleschi et al. 2007) and confer immunomodulatory effects. However, the present study reports that improved quality of life and reduced leg movements during sleep were concomitantly associated with increased serum BDNF levels after intervention in patients receiving placebo, highlighting the influence of the “placebo effect” as part of therapeutic interventions in nutrition and dietetic practice (Staudacher et al. 2017). Moreover, previous studies have highlighted the significance of accounting for the “placebo effect” when assessing the impact of experimental supplements on the progression of multiple sclerosis. Factors such as social support and external influences can affect patients' perceived quality of life. Notably, participation in a clinical trial alone—regardless of group assignment—may produce beneficial effects on disease activity (La Mantia et al. 1996). Likewise, the ongoing follow‐up and support provided by the research team can enhance MS patients' perception of their quality of life (Heesen and Cohen 2014).

Second, we measured the BDNF in the serum sample, not the tissue of MS patients. A positive association has been found between peripheral and central BDNF concentrations in previous studies, indicating that the BDNF level in the blood can be an indicator of the BDNF level in the central nervous system (Liu et al. 2023). Third, we drew blood after the early morning hours. Bus et al. found attenuated serum BDNF levels when blood was drawn later in the morning (Bus et al. 2011). However, diurnal variation has been reported for plasma BDNF levels, but not for serum BDNF levels (Piccinni et al. 2008). Fourth, a previous study found that a 24‐week exercise program significantly increased BDNF levels in patients with RRMS compared to non‐exercising MS patients (Wens et al. 2016). In the current study, baseline levels of high‐ and moderate‐intensity weekly physical activity were approximately 1.7 and 3 times higher, respectively, in the control group compared to the intervention group. Although these differences were not statistically significant, the higher physical activity levels in the control group may have contributed to elevated serum BDNF concentrations, potentially influencing the outcomes.

Fifth, we observed a higher dietary intake of poultry meat in the control group compared to the intervention group. Poultry is recognized as a modest dietary source of long‐chain omega‐3 polyunsaturated fatty acids, with an estimated global availability of approximately 0.62 mg of LC‐PUFA per day (Cartoni Mancinelli et al. 2022). To account for this difference, we applied Quade's ANCOVA, adjusting for baseline BDNF serum levels and poultry intake as covariates. After adjustment, the change in BDNF serum levels from baseline to week 12 did not differ significantly between groups.

### 4.2 High Sensitivity‐CRP Serum Levels and n‐3 Supplementation in MS

The results of our research indicated no significant differences between the intervention and control groups in hs‐CRP serum levels before and following the intervention. Furthermore, administering 1000 mg of omega‐3 fatty acid supplements twice daily, alongside a placebo of 1000 mg of oral paraffin soft capsules twice daily for 12 weeks, did not result in any significant changes in hs‐CRP serum concentrations in either group. In addition, omega‐3 fatty acids supplementation had no impact on hs‐CRP serum levels, with no significant differences observed between the two groups for this metric. The findings also revealed that the average changes in hs‐CRP serum levels were 1.5 times greater in female patients with MS compared to their male counterparts. Further analysis of our data assessing the influence of n‐3 PUFA supplementation on cardiovascular disease risk related to hs‐CRP suggested that the intervention with n‐3 PUFA resulted in a transition from high to intermediate risk within the intervention group. It indicates that while n‐3 PUFA supplementation seemed to lower high‐risk hs‐CRP levels, it did not significantly outperform the control group concerning overall risk distribution.

Numerous studies have looked into the effects of omega‐3 fatty acid supplementation on hs‐CRP, an inflammation marker (Elisia et al. 2022; Megawati et al. 2023; Mortazavi et al. 2018; Taha et al. 2022). Although a search of the literature did not uncover any studies that specifically focused on the influence of n‐3 supplementation on hs‐CRP serum levels in MS patients, this study is, to our knowledge, the first to evaluate the effect of n‐3 supplementation on hs‐CRP serum levels in this patient group. Findings from other neurodegenerative disorders have produced inconsistent results (Detopoulou et al. 2024; Freund‐Levi et al. 2009; Lin et al. 2022). One clinical trial assessed the impact of omega‐3 fatty acids on inflammatory markers in individuals with mild to moderate Alzheimer disease. After 6 months of supplementation, no significant alterations were noted in hs‐CRP levels or other inflammatory markers in either cerebrospinal fluid or plasma (Freund‐Levi et al. 2009). A randomized placebo‐controlled trial investigated the effects of omega‐3 fatty acids on blood‐based biomarkers in subjects with mild cognitive impairment and mild to moderate Alzheimer disease across 24 months. The study identified a significant reduction in the chemokine CCL4 following supplementation with EPA or a mixture of EPA and DHA. However, specific changes in hs‐CRP levels were not reported (Lin et al. 2022). Research on the concurrent intake of omega‐3 fatty acids and vitamin E supplementation in patients with Parkinson disease showed improvements in the UPDRS (unified Parkinson disease rating scale) alongside decreases in hs‐CRP levels (Taghizadeh et al. 2017). These results imply possible anti‐inflammatory effects; however, further investigation is needed to substantiate these findings. Consistent with our results, a double‐blind, placebo‐controlled clinical trial tested whether supplementation of 1400 mg of combined EPA and DHA over 18 weeks would reduce hs‐CRP levels. However, the study found no significant impact on inflammatory markers like hs‐CRP in a healthy cohort (Muldoon et al. 2016).

In general, although certain research suggests that omega‐3 fatty acids supplementation could affect inflammatory markers in neurodegenerative conditions (Detopoulou et al. 2024; Freund‐Levi et al. 2009; Thomas et al. 2015), the evidence regarding its effect on hs‐CRP levels has remained inconclusive. Further research is necessary to clarify these relationships.

In summary, while direct studies on the effect of n‐3 supplementation on hs‐CRP levels in MS patients are lacking, existing research in other populations indicates a discrepancy. This variability may relate to the metabolic characteristics of the study population, suggesting a possible interaction between diet and phenotype (Browning 2003). Additional studies are needed to confirm these benefits specifically in the context of MS.

Further analysis of our data using simple linear regression revealed that female MS patients exhibited 1.5 times more changes in hs‐CRP serum levels than their male counterparts. Some studies have shown that the mean level of CRP in women's serum can be higher than in men (Arcari et al. 2008; Khera et al. 2005, 2009). This difference can be related to various factors, including sex hormones like estrogen, which can affect CRP levels. For example, the use of hormone replacement therapy can raise CRP levels in women (Gaskins et al. 2012). Numerous studies have reported fluctuations in serum CRP levels across the menstrual cycle, likely influenced by endogenous reproductive hormones. These findings support the notion that natural estradiol may exert anti‐inflammatory effects. Consequently, it is important to standardize CRP measurements based on the menstrual cycle phase in women of reproductive age (Blum et al. 2005; Capobianco et al. 2010; Gaskins et al. 2012; Jilma et al. 1997; Wander et al. 2008; Wunder et al. 2006). In addition, women have a higher body fat percentage than men, and visceral fat can lead to increased CRP levels since adipose tissue can enhance the production of inflammatory cytokines. Women have consistently had higher hs‐CRP levels than men. The strongest hs‐CRP determinant is BMI in women and waist circumference in men (Arcari et al. 2008). Also, hormonal fluctuations during the menstrual cycle may affect CRP levels (Gaskins et al. 2012; Wander et al. 2008). Finally, conditions characterized by autoimmune responses and inflammation, particularly prevalent in women like systemic lupus erythematosus and rheumatoid arthritis, may elevate CRP levels (Enocsson et al. 2021; Pope and Choy 2021).

The study's findings indicate that omega‐3 fatty acids supplementation has no significant benefits on the evaluated primary and secondary outcomes in MS, echoing the lack of significant differences reported in other clinical trials regarding physical performance and cognitive function (Bischoff‐Ferrari et al. 2020; Castellanos‐Perilla et al. 2024), disease activity, relapse rates, disability progression, fatigue, quality of life, and safety of the treatment, both alone and with interferon beta‐1a (Torkildsen et al. 2012). For instance, 402 subjects with mild to moderate Alzheimer disease taking DHA 2000 mg daily for 18 months were studied by Quinn et al. (2010) and reported no modifications in brain atrophy. Despite supporting evidence from various clinical trials, the unpredictable outcomes related to n‐3 PUFAs suggest that randomized placebo‐controlled studies are essential to clarify their potential role in preventing or managing neurodegenerative disorders, while accounting for factors such as genetic polymorphisms and sex differences that may influence results (Castellanos‐Perilla et al. 2024).

In line with the findings of other clinical trials and the results of the present study, although n‐3 supplements offer an alternative source of dietary n‐3 PUFAs, the conflicting results from clinical trials raise questions about the determinants of their success (Castellanos‐Perilla et al. 2024). Therefore, randomized, placebo‐controlled trials are required to understand the potential impact of n‐3 supplementation in the treatment or even prevention of neurological diseases. However, any effect is likely to be modest and develop gradually, so trials should be of sufficient duration and sample size.

On the other hand, the metabolism of n‐3 PUFAs is a complex challenge. These PUFAs can follow complex metabolic pathways with different rates of incorporation into tissues, and genetic polymorphisms can influence the results of supplementation. In addition, gender‐related differences should not be ignored. The differences in their metabolism and omega‐3 fatty acids synthesis should be taken into account when designing and analyzing the results of supplementation trials. Finally, whether 2000 mg/d or a lower dose of n‐3 PUFAs affects other measures of neurotrophic factors and inflammation warrants investigation.

A key strength of this study is its sample size, which was adequate to identify small and medium treatment effects related to both biochemical and clinical disease activity.

The main limitations of this study were its high dropout rate (19% overall, including 32% in the intervention group), which reduced statistical power and affected the generalizability of the findings. The absence of postintervention data for participants who dropped out prevented the use of data imputation. Although sensitivity analyses indicated minimal bias, future studies should incorporate imputation techniques—such as multiple imputation—to address missing data more effectively.

The observed dropout rate, which exceeded that reported in a comparable study (Torkildsen et al. 2012), may be partially attributed to the study's inclusion and exclusion criteria. Specifically, 3 of the 11 participants in the intervention group were withdrawn due to pregnancy, tuberculosis, and shingles. Additionally, more than half of the early discontinuations were due to participants' unwillingness to continue or refusal to undergo blood sampling. Notably, around one‐third of all dropouts occurred within the first month of the trial. The early increase in dropout rates may have also been caused by the strict exclusion criteria, which required participants to be withdrawn if they missed even one of the four post‐baseline visits. Notably, medication intolerance accounted for only 1% of all withdrawals. In total, 55 out of the 68 randomized participants (80%) completed the study and attended the 12‐month follow‐up visit. Unfortunately, comparisons between those who withdrew and those who completed the trial were not possible due to limited data; participants who discontinued were not allowed to submit test results at the time of withdrawal.

No additional analyses were performed to compare participants who completed the study with those who withdrew. It is possible that individuals who dropped out faced greater difficulties adhering to the supplement regimen or attending scheduled visits at the neurological center. Conversely, those who completed the study may have had higher treatment compliance. As a result, the elevated dropout rate could compromise both the generalizability and statistical power of the study findings.

A second limitation is the baseline imbalance in hs‐CRP risk categories (*p* = 0.012), which may have introduced residual confounding despite statistical adjustments. This issue is further compounded by the lack of standardization of hs‐CRP measurements according to menstrual cycle phase in women of reproductive age. Given that hs‐CRP levels fluctuate throughout the menstrual cycle in normally ovulating women, these variations should be accounted for in future investigations to improve the accuracy of serum hs‐CRP assessment. The cycle‐dependent nature of hs‐CRP highlights the precise timing of blood sampling for clinical and research purposes (Wunder et al. 2006). To address potential imbalances and reduce confounding, future studies should aim for well‐balanced baseline characteristics or utilize larger sample sizes that allow for advanced statistical approaches, such as propensity score matching.

A third limitation is the use of per‐protocol analysis instead of an intention‐to‐treat approach, which may have led to an overestimation of treatment effects by excluding participants who dropped out. To enhance the validity and reliability of results, future studies should adopt intention‐to‐treat analyses and apply appropriate imputation techniques to address missing data.

We conducted a 12‐week intervention using a daily dose of 2000 mg/d based on parameters established in previous clinical trials (Ziaei et al. 2024). The lack of observed effects on BDNF and hs‐CRP may suggest that omega‐3 supplementation, at this dosage and duration, is not effective in individuals with MS. However, variations in dosage and intervention length could yield different outcomes, as suggested by Liu et al. (2023) and Sohouli et al. (2023), indicating a need for further research in this area.

## 5 Conclusion

Administration of 1000 mg of omega‐3 fatty acid supplements twice a day, along with a placebo group receiving 1000 mg of oral paraffin soft capsules twice a day for 12 weeks, significantly increased serum BDNF levels in both the intervention and control groups compared to baseline. The observed rise in BDNF levels among placebo recipients highlights the potential influence of the placebo effect. Elements such as social support and the structured follow‐up and encouragement provided by the research team may have improved participants' perceived quality of life, thereby positively affecting disease‐related outcomes, even in those receiving a placebo. Moreover, the analysis showed that omega‐3 supplementation, as well as variables such as sex, age, education, BMI, EDSS score, chronic fatigue subscales, and physical activity levels, had no significant impact on changes in BDNF serum concentrations.

Our research results showed no significant differences in hs‐CRP serum levels between the intervention and control groups when assessed before and after the intervention. The results also showed that the average changes in hs‐CRP serum levels were 1.5 times higher in female MS patients than in their male counterparts. This disparity may be attributed to various factors such as hormones, body fat, menstrual cycles, and inflammatory conditions.

Furthermore, omega‐3 fatty acid supplementation in the intervention group was associated with a shift from high to intermediate hs‐CRP risk levels, suggesting a potential reduction in elevated inflammation markers. However, this effect did not significantly surpass that observed in the control group in terms of overall risk distribution. These findings underscore the need for additional clinical trials exploring lower dosages to define optimal supplementation strategies better They also highlight the importance of standardizing CRP measurements according to menstrual cycle phases in women of reproductive age to ensure accurate assessment.

## Author Contributions

**Sahar Golabi:** resources (equal), visualization (equal), writing – review and editing (equal). **Tahereh Robahat:** methodology (equal). **Nastaran Madjdinasab:** investigation (equal), resources (equal), validation (equal). **Naser Kamyari:** data curation (equal), formal analysis (equal), validation (equal), writing – review and editing (equal). **Mahshid Naghashpour:** conceptualization (equal), funding acquisition (equal), methodology (equal), project administration (equal), resources (equal), supervision (equal), visualization (equal), writing – original draft (equal).

## Ethics Statement

To comply with ethical considerations, patients continued their routine MS‐modifying drugs during the study. This study was approved by the Research Ethics Committees of the Abadan University of Medical Sciences with the Ethics Code IR.ABADANUMS.REC.1399.006 on 2020‐04‐11 and has therefore been performed in accordance with the ethical standards laid down in the 1964 Declaration of Helsinki and its later amendments. It was also approved by the Iranian Registry of Clinical Trials (IRCT) with the IRCT registry code IRCT20240404062003N1 on 2024‐07‐28.

## Consent

Written informed consent was obtained from all study participants.

## Conflicts of Interest

The authors declare no conflicts of interest.

## Data Availability

The data that support the findings of this study are available on request from the corresponding author.
